# Colorectal cancer prevention by non-steroidal anti-inflammatory drugs: effects of dosage and timing

**DOI:** 10.1038/sj.bjc.6690651

**Published:** 1999-09

**Authors:** J-P Collet, C Sharpe, E Belzile, J-F Boivin, J Hanley, L Abenhaim

**Affiliations:** Centre for Clinical Epidemiology and Community Studies, Sir Mortimer B. Davis–Jewish General Hospital, 3755 Chemin de la Côte-Ste-Catherine, Montréal, H3T 1E2, Canada; Joint Departments of Epidemiology and Biostatistics and of Occupational Health, McGill University, Montréal, Canada

**Keywords:** colorectal cancer, non-steroidal anti-inflammatory drugs, epidemiology, case–control studies

## Abstract

Epidemiological studies show that non-steroidal anti-inflammatory drugs (NSAIDs) reduce colorectal cancer incidence. We measured the rate ratio for colorectal adenocarcinoma according to dosage and the timing of exposure by means of a case–control study, nested in a non-concurrent cohort linkage study, using the population of beneficiaries of the Saskatchewan Prescription Drug Plan from 1981 to 1995 with no history of cancer since 1970 as the source population. Four controls per case, matched on age and gender and alive when the case was diagnosed, were randomly selected. Dispensing rates, calculated over successive time periods, characterized NSAID exposure. We accrued 3844 cases of colon cancer and 1971 cases of rectal cancer. For colon cancer a significant trend towards a decreasing rate ratio was associated with increasing exposure during the 6 months preceding diagnosis (P-trend = 0.002). For both cancers, significant trends were associated with exposure 11–15 years before diagnosis (colon: P-trend = 0.01; rectum: P-trend = 0.0001). At the highest exposure levels the rate ratio for colon cancer was 0.57 (95% confidence interval (CI) 0.36–0.89); for rectal cancer it was 0.26 (95% CI 0.11–0.61). No protection was associated with exposure during other periods. The timing of NSAID use must be considered in planning intervention trials to prevent colorectal cancer. There may be a 10-year delay before any preventive effect will appear. © 1999 Cancer Research Campaign


*British Journal of Cancer* (1999) **81**(1), 62–68
© 1999 Cancer Research Campaign
Article no. bjoc.1999.0651

### Colorectal cancer prevention by non-steroidal anti-inflammatory drugs: effects of dosage and timing

**J-P Collet[1,2], C Sharpe[1,2], E Belzile[1], J-F Boivin[1,2], J Hanley[2] and L Abenhaim[1,2]**

[1]Centre for Clinical Epidemiology and Community Studies, Sir Mortimer B. Davis–Jewish General Hospital, 3755 Chemin de la Côte-Ste-Catherine, Montréal, Canada H3T 1E2; [2]Joint Departments of Epidemiology and Biostatistics and of Occupational Health, McGill University, Montréal, Canada

Summary Epidemiological studies show that non-steroidal anti-inflammatory drugs (NSAIDs) reduce colorectal cancer incidence. We measured the rate ratio for colorectal adenocarcinoma according to dosage and the timing of exposure by means of a case–control study, nested in a non-concurrent cohort linkage study, using the population of beneficiaries of the Saskatchewan Prescription Drug Plan from 1981 to 1995 with no history of cancer since 1970 as the source population. Four controls per case, matched on age and gender and alive when the case was diagnosed, were randomly selected. Dispensing rates, calculated over successive time periods, characterized NSAID exposure. We accrued 3844 cases of colon cancer and 1971 cases of rectal cancer. For colon cancer a significant trend towards a decreasing rate ratio was associated with increasing exposure during the 6 months preceding diagnosis (P-trend = 0.002). For both cancers, significant trends were associated with exposure 11–15 years before diagnosis (colon: P-trend = 0.01; rectum: P-trend = 0.0001). At the highest exposure levels the rate ratio for colon cancer was 0.57 (95% confidence interval (CI) 0.36–0.89); for rectal cancer it was 0.26 (95% CI 0.11–0.61). No protection was associated with exposure during other periods. The timing of NSAID use must be considered in planning intervention trials to prevent colorectal cancer. There may be a 10-year delay before any preventive effect will appear.

**Keywords:** colorectal cancer; non-steroidal anti-inflammatory drugs; epidemiology; case–control studies

Evidence that non-steroidal anti-inflammatory drugs (NSAIDs) prevent colorectal adenocarcinoma comes from numerous animal (Pollard and Luckert, 1981; Birkenfeld et al, 1987) and human studies, which include cohort studies, case–control studies, and one randomized clinical trial. Most have shown that NSAIDs have protective effects with rate ratios from 0.7 to 0.2. However, one cohort study found an excess risk in the elderly exposed to aspirin (Paganini-Hill et al, 1989) and the only randomized clinical trial (Gann et al, 1993) showed no effect after 5 years of follow-up.

Case–control studies have been limited by poor recall of exposure. Recently, however, Pelleg et al (1996) used a hospital drug database to assess exposure. Although they found a strong dose-dependent protective effect, the results were difficult to interpret because the study was hospital-based and the controls were selected randomly without considering their admission diagnoses.

Cohort studies may have been less prone to problems of exposure assessment. In most of these studies, however, exposure was limited to an estimate of exposure at entry based on data obtained by interview or self-administered questionnaire (Paganini-Hill et al, 1989; Thun et al, 1991; Gridley et al, 1993; Schreinemachers and Everson 1994). Only two collected information repeatedly to study the effects of dose and duration of exposure to NSAIDs (Giovannucci et al, 1994*c*, 1995). Giovannucci et al (1995) found a protective effect (rate ratio = 0.63, 95% confidence interval (CI) 0.42–0.94) among women who took ≥ 2 aspirin tablets per week for ≥ 10 years. They did not find a dose–response relationship and concluded that benefit may not appear until after a decade of regular use.

Do similar exposures at different times have similar or different effects? To answer this question in relation to the prevention of colorectal cancer by drug exposure the combined induction and latent periods for the drugs studied must be determined, i.e. the time which must pass before the preventive effect of an exposure will become detectable. Failure to do so will dilute the measured effect of exposure. For example, cumulative exposure may include both aetiologically relevant remote use that could have an effect on disease incidence at a specific time, as well as aetiologically irrelevant recent use that could not have any effect because the induction and latent periods have not yet elapsed (Rothman, 1981).

To study the effects of dose and the timing of NSAID use we carried out a population-based case–control study, nested in a non-concurrent cohort linkage study in Saskatchewan, using pre-recorded data collected routinely by the Saskatchewan Cancer Agency (SCA) and the Saskatchewan Prescription Drug Plan (SPDP; operating since 1975). These agencies provide universal coverage to the Saskatchewan population. This offered us an opportunity to carry out a study with minimal potential for selection and recall bias and gave us drug exposure histories of sufficient length to determine the effects of dose and the timing of NSAID use on the risk of colorectal cancer.

*Received 3 September 1998*
*Revised 24 March 1999*
*Accepted 12 April 1999*

*Correspondence to:* J-P Collet

### SUBJECTS AND METHODS

### Definitions of study populations

We defined a source population for the study from which we sampled cases and controls. It was the open population of men and women aged 35 years or older who were eligible to receive benefits from the SPDP during 1981 to mid-1995, with no history of cancer since 1970 other than non-melanoma skin cancer or carcinoma in situ of the cervix. Earlier cancer diagnoses were unavailable, because the records of the SCA were not computerized prior to 1970.

Subjects entered the source population on 1 January 1981 if they were aged 35 or older, or on their 35th birthday, or on the date of immigration into the province if aged 35 years or older, whichever occurred latest. Subjects left the source population on 30 June 1995, on the date of diagnosis of colorectal cancer or of death, or on the date of emigration, whichever occurred first.

The SPDP pays for prescription drugs for 94% of the Saskatchewan population (1.01 million people in mid-1991) (Rawson et al, 1992). Military personnel and aboriginals, who are covered by federal agencies, are excluded. Immigrants become eligible to benefit 3 months after arriving, which is recorded as the coverage initiation date. Emigrants lose their eligibility 3 months after leaving. Emigration from a population similar to that of this study was estimated to be < 0.8% per year (Risch and Howe, 1994). Every person eligible to receive benefits is issued with a plastic card on which is stamped a unique personal identification number which can be used to identify the same individual over time in the same database and across different databases, including that of the SCA. Until the end of 1990 the personal identification number consisted of eight digits; since 1991 it consisted of nine digits, one of them representing the sum of other digits so that the validity of the entire identification number can be checked (Rawson et al, 1992). The nine digit identification numbers are also encoded in a magnetic strip on beneficiaries' plastic cards, so that services can be registered electronically. The accuracy of the identifying information used by the SPDP exceeds 99.99% and the accuracy of the recorded prescription information exceeds 99% (Risch and Howe, 1994).

The cases were defined as subjects in the source population who were diagnosed with histologically proven adenocarcinoma of the colon or rectum and reported to the SCA. Registration of cancer cases was likely to be nearly complete because physicians' claims for services are paid only for registered cases and copies of all malignant pathology reports are sent to the SCA. These two mechanisms of notification cover approximately 98% of all new cancer cases diagnosed within the province; an additional 1–2% of cases are discovered through death certificates received biweekly (Parkin et al, 1997). To be included in our study cases must have been eligible to benefit from the SPDP for at least 5 years before diagnosis; this was to ensure that sufficiently long records of their drug use, if any, would be available for analysis. Hereafter, the date of diagnosis will be designated as the 'index date'.

Potential controls were defined as the subjects in the source population of the same age (expressed as an integral number of years) and gender as the case to whom they were individually matched and who were alive on the date upon which the case was diagnosed and therefore at risk of developing colorectal cancer. The date of diagnosis for each case was assigned to each of the matched potential controls as their index date, thereby matching cases and controls on sampling time as well (Rothman and Greenland, 1998). Like the cases, the controls must have been eligible to benefit from the SPDP for at least 5 years before the index date.

### Linkage methods

Information pertaining to individuals registered in the databases of the SPDP and the SCA was linked by matching personal identifiers. Subjects accrued from 1 January 1981 until 31 December 1990 had their records linked on the basis of an exact match of three variables: the eight digit personal identification number, the year of birth (±2 years, because of inconsistent reporting of the year of birth by some very old subjects), and gender. Subjects accrued after 1 January 1991 had their records linked on the basis of an exact match of the nine-digit personal identification number.

### Methods of selection of cases and controls

Computerized lists of all cases of histologically proven adenocarcinoma of the colon or rectum aged 35 years or more at diagnosis with no history of cancer since 1970, other than non-melanoma skin cancer and carcinoma in situ of the cervix, reported to the SCA as having been diagnosed from 1 January 1981 until 30 June 1995, were obtained. These lists from the SCA were cross-referenced against the database of the SPDP using the linkage procedures described above. Cases whose coverage initiation dates for the SPDP preceded their index dates by less than 5 years were then excluded. In selecting the cases we were blind to the nature of which drugs they took, if any, during their exposure histories.

For each selected case, the SPDP database was searched to compile a separate computerized list of individually matched potential controls. All the potential controls on each list were of the same age and gender as the case and were alive on the case's index date. Potential controls whose coverage initiation dates for the SPDP preceded their index dates by less than 5 years were then excluded from each list. As with the cases, we were blind to the nature of which drugs they took, if any, during their exposure histories. These lists were then cross-referenced against the SCA database using the linkage procedures described above to determine the date of diagnosis of every cancer, if any, diagnosed in each potential control back to 1970. Subjects with cancer diagnoses, other than non-melanoma skin cancer and carcinoma in situ of the cervix, that occurred before the index date of the case to whom they were matched were then excluded from each list. Each list was then sampled randomly without replacement so that four controls per case were selected. This method of selecting controls is known as risk-set sampling (Rothman and Greenland, 1998).

### Drug exposure data

For all subjects drug exposure data were obtained from the SPDP database which provided information for the period between the index date back to January 1 1976 or the coverage initiation date, whichever was later. The drug exposure histories ranged from 5 to 19.5 years in length.

For each prescription for NSAIDs dispensed to a subject as an outpatient, the following information was recorded: the subject's unique identification number, the dispensing date, the class and identity of the drug according to the American Hospital Formulary System classification, the number of doses dispensed, and the

© 1999 Cancer Research Campaign strength (mg per pill). The number of pills per day recommended and the duration of treatment were not available.

Information was also obtained about other drugs that could confound the exposure–disease relationship, e.g. drugs used in the treatment of inflammatory bowel disease and oestrogens.

No data on any drugs prescribed during hospitalizations or during the period 1 July 1987 to 1 January 1989 were available, because the SPDP did not record the dispensing of drugs to individuals during that period. The database also lacks information on drugs given directly to patients by physicians as samples and drugs bought without prescriptions. Aspirin and ibuprofen can be bought without prescriptions, the latter since August 1989.

### Confidentiality

The extraction of the data from the electronic databases of the SCA and the SPDP was carried out by employees of the SCA and Saskatchewan Health. The data delivered for analysis were devoid of any information that could be used to identify anyone. No subjects were contacted to obtain information. The study was approved by the ethics committees of the Sir Mortimer B Davis–Jewish General Hospital, Montreal, the Cross Agency Study Committee of Saskatchewan Health, Regina, and the Internal Review Board of the SCA.

### Data analysis

We analysed exposure to NSAIDs as a class rather than exposure to individual drugs, recognizing that NSAIDs in general reduce the risk of colorectal cancer (Berkel et al, 1996). This strategy provided the analysis with sufficient power to study the effects of timing of exposure because the numbers of subjects with histories of use of any one drug were relatively small.

To study the effects of the timing of exposure we decided *a priori* to divide time preceding the index date into successive 5-year periods. The period immediately preceding the index date was divided further into two 6-month periods and the preceding 4-year period. The periods were: 1–6 months, 7–12 months, 2–5 years, 6–10 years and 11–15 years.

Exposure was characterized in terms of average rates of dispensing NSAIDs, calculated from the amounts dispensed over the periods described. Three different methods of calculating dispensing rates were used and all produced similar patterns of results. The first two methods were based on the number of either prescriptions or pills dispensed per year; the results of analyses using these methods are not shown for brevity. The third method was based on the proportion of the maximum recommended daily dose of each $NSAID_i$ dispensed ($p_i$ = average mg $day^{-1}$ dispensed ÷ maximum mg $day^{-1}$ recommended) (Carson et al, 1987) and was calculated for each different NSAID used during each period of time. The sum of the proportions, i.e. $\Sigma p_i$ for all the NSAIDs used during a period, was considered as the measure of exposure. This method distinguished non-users from low levels users ($0 < \Sigma p_i \leq 0.1$), from medium level users ($0.1 < \Sigma p_i \leq 0.3$), from high level users ($\Sigma p_i > 0.3$). For some high level users $\Sigma p_i$ exceeded 1.0. These categories were determined by examining frequency distributions of $\Sigma p_i$. The usual maximum daily dose for each NSAID was taken from the manufacturers' recommendations (*Compendium of Pharmaceuticals and Specialties*, 1995). The drugs, their recommended maximum daily doses, and the total number of prescriptions of each drug dispensed between the date on which subjects first became eligible to benefit from the SPDP and the index date for all subjects are shown in Table 1.

**Table 1** Listed are the NSAIDs dispensed to all cases of colon cancer, rectal cancer and controls between the date on which subjects first became eligible to receive benefits from the SPDP and each subject's index date. Beside each drug is the recommended maximum daily dose and the total number of prescriptions of each drug dispensed (N)

| Drug | Usual maximum daily dose (mg $day^{-1}$) | N (Rx) |
|---|---|---|
| Aspirin | 3900 | 76 867 |
| Diclofenac | 150 | 16 451 |
| Diflunisal | 1000 | 4666 |
| Fenoprofen | 2400 | 10 906 |
| Floctafenene | 1200 | 605 |
| Flurbiprofen | 200 | 577 |
| Ibuprofen | 1200 | 39 619 |
| Indomethacin | 200 | 49 807 |
| Ketoprofen | 200 | 13 501 |
| Mefenamic acid | 1000 | 871 |
| Nabumetone | 2000 | 3 |
| Naproxen | 1000 | 35 251 |
| Phenylbutazone | 400 | 11 995 |
| Piroxicam | 20 | 26 938 |
| Sulindac | 400 | 15 368 |
| Tiaprofenic acid | 600 | 2482 |
| Tolmetin | 1800 | 1424 |
| Zomepirac | 400 | 349 |

The analysis was designed to deal with missing exposure information. Since the overall length of the drug exposure histories ranged from 5 to 19.5 years, for each subject there was information missing beyond a certain time in the past. In addition, subjects whose index dates occurred after 1 July 1987 had their histories interrupted by the 1.5-year period during which the SPDP database was incomplete and the use of most drugs was not recorded on an individual basis. If the drug exposure history for a period was missing or incomplete, due either to the date of the subject's enrolment in the SPDP or to the 1.5-year period of missing information, the subject was assigned to a separate category designated 'other', distinct from the referent and the categories of exposure (Breslow and Day, 1980; Miettinen, 1985). This category can be considered to represent an indeterminate level of exposure. Because the maximum length of the exposure histories was 19.5 years, we truncated the exposure histories at 15 years before the index date – otherwise, all subjects with longer exposure histories would be categorized in the 'other' category for the preceding 5-year period.

This approach allowed us to retain each subject in the analysis, the aim of which was to estimate the effects of similar exposures at different times over a 15-year span, rather than exclude subjects with missing information. Even though the exposure histories of many subjects were less than 15 years long and many were interrupted by the 1.5-year gap, subjects contributed information to the analysis of the effects of NSAID exposure during the periods for which we had complete exposure information, and no information to the periods for which we lacked complete information. For example, subjects who immigrated to Saskatchewan 12.5 years prior to their index date would contribute no information to the analysis of the effects of exposure 11–15 years before the index date, but would contribute information to the periods closer to the index date, provided they were not interrupted by the 1.5-year gap.

© 1999 Cancer Research Campaign

We calculated odds ratios to estimate incidence density ratios (rate ratios) with conditional logistic regression to account for the individual matching on age, gender and index date (Breslow and Day, 1980) by means of the SAS PHREG procedure (SAS Institute, 1992). The results are presented with 95% CIs. The statistical model that we used to relate the rate ratios to the drug exposure history over time was:

$$\exp(\beta_1 X_{1\text{–}6\text{ mos}} + \beta_2 X_{7\text{–}12\text{ mos}} + \beta_3 X_{2\text{–}5\text{ yrs}} + \beta_4 X_{6\text{–}10\text{ yrs}} + \beta_5 X_{11\text{–}15\text{ yrs}})$$

where the values of $\beta_i$ represented the regression coefficients, and the values of $X_i$ represented drug exposure during the successive periods of time preceding the index date. This approach was based on Miettinen's recognition that exposures during 'different time periods represent separate determinants … mutually confounded and thus requiring joint representation in the same model' (Miettinen, 1985). Since NSAIDs are used for chronic conditions and exposure during one period could be associated with exposure in another, mutual adjustment for successive periods of exposure was required.

The rate ratios for a particular period calculated in this way represented the ratio of the incidence of disease among those exposed to NSAIDs during that period to the incidence of disease among those unexposed during that period. The mutual adjustment for successive periods of exposure provided by the statistical model permitted us to estimate the effect of varying NSAID exposure during a given period while keeping the pattern of NSAID exposure during all the other periods identical.

Exposure was represented by categorical variables. There were five categories of exposure: unexposed (the referent), low, medium, high and 'other'. We did not use $\Sigma p_i$ as a continuous variable because it was not always linearly related to the log of the rate ratio.

Testing for trends was carried out by representing the categories of exposure in the models with ordinal variables, considered as continuous, and examining the significance of the coefficients with a $\chi^2$ test (Breslow and Day, 1980). *P*-values for interaction were derived from likelihood ratio tests comparing nested models with and without interaction terms. We used $P < 0.05$ (two-sided) as the criterion of statistical significance.

### RESULTS

We accrued 3844 colon cancer cases (1925 males) with 15 373 controls and 1971 rectal cancer cases (1230 males) with 7882 controls. The mean age of the colon cancer cases was 70.8 years (11.6 standard deviation (s.d.)); for the rectal cancer cases it was 69.4 years (11.6 s.d.).

**Table 2** Rate ratios for colon cancer and for rectal cancer according to NSAID exposure by time period before diagnosis, with adjustment for exposure during the other time periods

| | | Colon Cancer | | | | Rectal Cancer | | | |
|---|---|---|---|---|---|---|---|---|---|
| Period before diagnosis | Average daily dose | Cases *n* = 3844 | Controls *n* = 15373 | RR[a] | 95% CI | Cases *n* = 1971 | Controls *n* = 7882 | RR[a] | 95% CI |
| 1–6 months | 0 | 2673 | 10214 | 1.00 | Referent | 1372 | 5373 | 1.00 | Referent |
| | $0 < \Sigma p_i \leq 0.1$ | 235 | 871 | 1.06 | 0.91–1.24 | 102 | 401 | 1.01 | 0.80–1.28 |
| | $0.1 < \Sigma p_i \leq 0.3$ | 220 | 1012 | 0.88 | 0.75–1.04 | 115 | 473 | 0.99 | 0.79–1.25 |
| | $\Sigma p_i > 0.3$ | 167 | 1080 | 0.69 | 0.56–0.86 | 97 | 495 | 0.80 | 0.59–1.08 |
| | *P* (trend) | | | | 0.002 | | | | 0.26 |
| | Other | 549 | 2196 | – | – | 285 | 1140 | – | – |
| 7–12 months | 0 | 2667 | 10175 | 1.00 | Referent | 1365 | 5387 | 1.00 | Referent |
| | $0 < \Sigma p_i \leq 0.1$ | 197 | 816 | 0.93 | 0.79–1.10 | 101 | 374 | 1.12 | 0.88–1.41 |
| | $0.1 < \Sigma p_i \leq 0.3$ | 233 | 1014 | 0.97 | 0.83–1.15 | 123 | 510 | 1.01 | 0.81–1.27 |
| | $\Sigma p_i > 0.3$ | 191 | 1144 | 0.85 | 0.68–1.05 | 105 | 503 | 0.93 | 0.68–1.25 |
| | *P* (trend) | | | | 0.08 | | | | 0.99 |
| | Other | 556 | 2224 | – | – | 277 | 1108 | – | – |
| 2–5 years | 0 | 1102 | 4177 | 1.00 | Referent | 648 | 2446 | 1.00 | Referent |
| | $0 < \Sigma p_i \leq 0.1$ | 855 | 3357 | 0.99 | 0.89–1.10 | 420 | 1842 | 0.87 | 0.76–1.00 |
| | $0.1 < \Sigma p_i \leq 0.3$ | 193 | 922 | 0.90 | 0.75–1.08 | 104 | 421 | 1.03 | 0.80–1.32 |
| | $\Sigma p_i > 0.3$ | 106 | 566 | 0.97 | 0.76–1.24 | 68 | 250 | 1.29 | 0.94–1.78 |
| | *P* (trend) | | | | 0.41 | | | | 0.80 |
| | Other | 1588 | 6351 | – | – | 731 | 2923 | – | – |
| 6–10 years | 0 | 766 | 3022 | 1.00 | Referent | 412 | 1564 | 1.00 | Referent |
| | $0 < \Sigma p_i \leq 0.1$ | 808 | 3061 | 1.07 | 0.96–1.20 | 367 | 1477 | 0.97 | 0.83–1.14 |
| | $0.1 < \Sigma p_i \leq 0.3$ | 135 | 638 | 0.92 | 0.75–1.13 | 67 | 282 | 0.92 | 0.69–1.24 |
| | $\Sigma p_i > 0.3$ | 69 | 362 | 0.94 | 0.71–1.24 | 28 | 155 | 0.77 | 0.50–1.18 |
| | *P* (trend) | | | | 0.75 | | | | 0.31 |
| | Other | 2066 | 8290 | 0.88 | 0.61–1.26 | 1097 | 4404 | 0.77 | 0.46–1.31 |
| 11–15 years | 0 | 590 | 2228 | 1.00 | Referent | 303 | 1047 | 1.00 | Referent |
| | $0 < \Sigma p_i \leq 0.1$ | 581 | 2218 | 1.01 | 0.88–1.15 | 235 | 1016 | 0.80 | 0.66–0.98 |
| | $0.1 < \Sigma p_i \leq 0.3$ | 67 | 386 | 0.73 | 0.55–0.96 | 22 | 125 | 0.63 | 0.39–1.01 |
| | $\Sigma p_i > 0.3$ | 23 | 181 | 0.57 | 0.36–0.89 | 6 | 82 | 0.26 | 0.11–0.61 |
| | *P* (trend) | | | | 0.01 | | | | 0.0001 |
| | Other | 2583 | 10360 | 0.87 | 0.64–1.18 | 1405 | 5612 | 0.95 | 0.62–1.45 |

[a]RRs (s) were calculated with conditional logistic regression because of matching for age, gender and index date. Exposure was categorized according to values of $\Sigma p_i$ (see Methods – Data Analysis) during the periods indicated. The 'other' category was for subjects with incomplete exposure information.

© 1999 Cancer Research Campaign

Three controls were excluded from the colon cancer study because their coverage initiation dates for the SPDP occurred after they entered the source population rather than before, which suggested that they may have left Saskatchewan and returned later, their emigration having been unrecorded. Two controls were excluded from the rectal cancer study for the same reason.

The rate ratio according to ever *vs* never dispensing of NSAIDs was 1.00 (95% CI 0.92–1.09) for colon cancer; 74.0% of both the cases and controls were exposed. For rectal cancer the rate ratio was 0.90 (95% CI 0.81–1.00); 69.6% of the cases and 71.7% of the controls were exposed.

Table 2 shows the results of analyses in which NSAID exposure was defined in terms of $\Sigma p_i$ calculated for each period, adjusting for the effects of exposure during the other periods. For colon cancer there were significant trends towards decreasing rate ratios associated with increasing NSAID exposure during the 6 months before the index date ($P$-trend = 0.002) and during the 11–15 years before the index date ($P$-trend = 0.01), with no significant trends in between. For rectal cancer there was also evidence of a significant trend towards decreasing rate ratios associated with increasing NSAID exposure during the 11–15 years before the index date ($P$-trend = 0.0001), but not during any other periods. The rate ratios associated with NSAID exposure did not depend on gender for either colon cancer ($P$ = 0.33, for interaction) or rectal cancer ($P$ = 0.35, for interaction).

The regression coefficients for the 'other' exposure category for the periods preceding the index date by ≤ 5 years could not be calculated because of insufficient variation: since cases and controls were matched for index date, if exposure information was missing for a case, due to the 1.5-year gap in the exposure histories beginning 1 July 1987, it was always missing for the matched controls, so they were all classified as 'other' and contributed no information to the matched analysis.

To determine if the dispensing of other drugs could confound the relationship between NSAID dispensing and the diagnosis of colon cancer or rectal cancer we began by determining if the dispensing of other drugs was also associated with the diagnosis of either cancer. We considered drugs dispensed to treat inflammatory bowel disease, which is associated with an increased risk of colorectal cancer (Potter et al, 1993), and oestrogens, which may be protective (Newcomb and Storer, 1995).

The dispensing of corticosteroids was not associated with the diagnosis of colon cancer or rectal cancer and so could not lead to confounding. However, the dispensing of either sulfasalazine or 5-aminosalicylic acid or both during the year preceding the index date was strongly associated with the diagnosis of colon cancer.

Very small numbers of women had exposure to oral contraceptives or progestins and there were no dose–response trends. However, appreciable numbers of women had received oestrogen replacement therapy and we detected negative associations between the dispensing of oestrogens during the 11–15 years before the index date and the diagnosis of both colon and rectal cancer.

To control possible confounding by exposure to sulfasalazine, 5-aminosalicylic acid, or oestrogen replacement therapy, we repeated the analyses shown in Table 2 after excluding all subjects with any exposure to either sulfasalazine or 5-aminosalicylic acid and including terms representing exposure to estrogens over time. The rate ratios associated with NSAID exposure were almost identical to those shown in Table 2 for both tumours.

### DISCUSSION

### Summary of findings

The dispensing of NSAIDs 11–15 years before the index date is associated with a dose-dependent reduction in the rate ratios for colon cancer and rectal cancer, using three different measures of exposure, one of which is shown in Table 2. For colon cancer, but not for rectal cancer, there was a trend towards a decreasing rate ratio with increasing dose associated with exposure during the 6 months preceding the index date.

### Representation of exposure

We represented exposure to NSAIDs as a class rather than as individual drugs. The analysis of the effects of individual drugs would have been limited by the small numbers of subjects exposed to each drug. In addition, animal experiments have shown that 'the beneficial effect of NSAIDs does not seem to be restricted to one particular drug' (Berkel et al, 1996). The underlying reason may be that all NSAIDs inhibit prostaglandin synthesis.

Considering exposure during different periods to represent separate determinants provided a simple way to represent the exposure history over time with multiple logistic regression, which allowed us to study the effects of the timing of exposure (Miettinen, 1985). The unexposed during each period were the referent for that period. This was justified by the fact that ingested NSAIDs are excreted and their pharmacological effects cease when use stops.

Our measures of exposure were based on average rates of dispensing computed over extended periods, usually 5 years, and focused on the timing of exposure. We did not study the effects of overall duration of exposure, since such analyses do not take the induction and latent periods of the disease into account and produce attenuated measures of effect, since they include periods of exposure irrelevant to the outcome (Rothman, 1981). Nor did we study the effects of duration of exposure within periods because we lacked information on the length of treatment periods. Since NSAID use is often intermittent (e.g. for the treatment of pain due to dysmenorrhea, headaches and injuries), we doubted that estimating duration of use would be valid.

Collinearity was not an issue in any of the analyses. None of the absolute values of the correlation coefficients between the regression coefficients in the analysis of Table 2 were larger than 0.52. The largest such value for the period 11–15 years before the index date was only 0.15. When exposures were considered separately (results not shown), the rate ratios tended to be similar but slightly more extreme than when included altogether in the models shown.

Since our measures of exposure were limited, being based solely on prescriptions dispensed to outpatients, it is likely that the subjects' actual consumption of NSAIDs differed somewhat from our estimates of exposure. On the one hand, it is unlikely that all the drugs dispensed were ingested: we may have overestimated exposure. On the other hand, we may have underestimated exposure, since we had no information about NSAIDs dispensed in hospitals or as samples in physicians' offices. These amounts were likely to be small relative to the amounts we used in calculating exposure. Nor did we have information about aspirin and ibuprofen bought over the counter: these amounts were also probably relatively small, since anyone needing to use appreciable amounts could request a prescription from a physician and the SPDP would pay. Subjects in the reference categories may have

© 1999 Cancer Research Campaign had low levels of exposure to either aspirin or ibuprofen or both.

If we overestimated NSAID exposure among those subjects classified as highly exposed, and underestimated NSAID exposure among those subjects classified as unexposed, then the slope of the dose–risk relationship that we observed would be less extreme than the true slope (MacMahon and Trichopoulos, 1996). It is possible, however, that the most highly exposed subjects may have ingested almost all of the NSAIDs dispensed, if they suffered chronic pain. If we accurately estimated overall NSAID exposure among the highly exposed and underestimated it among the non-exposed, then the true dose–risk curve would be shifted to the right relative to what we observed with an increased slope.

It is possible that the procedures used to link the database of the SPDP with that of the SCA could have resulted in some subjects having been assigned incorrect exposure histories and in some having been assigned an incorrect disease status, although we view these as unlikely possibilities due to the checks used to verify subjects' identities (Rawson et al, 1992). Such events would be expected to be rare, and occur randomly, independent of exposure or disease status. Their anticipated effect on our analysis would be to bias the RRs towards the null; that is, the true RRs would be more extreme than those we observed (Marshall et al, 1981; Brenner and Gefeller, 1993).

### Interpretation of results

The trend towards a decreasing rate ratio for colon cancer associated with increasing dose during the 6 months preceding the index date (Table 2) may have been due to a reduced rate of dispensing of NSAIDs among cases because of signs and symptoms caused by undiagnosed colon cancer, e.g. rectal bleeding, anaemia, abdominal pain. Physicians could have attributed these phenomena to NSAIDs and advised their patients to stop using them, or the cases could have stopped on their own. We have no information about the proportions of cases detected with and without symptoms. There is no formal population screening programme for colorectal cancer in Saskatchewan.

However, the trend could also be evidence of a growth-inhibiting effect of NSAIDs on colon tumours when they were large enough and growing rapidly enough to cause the symptoms that resulted in diagnosis. Such an effect could postpone diagnosis and explain our result. Evidence for such an effect in patients with diagnosed tumours comes from a randomized controlled trial of anti-inflammatory therapy in undernourished patients with several types of metastatic solid tumours (Lundholm et al, 1994). Patients were randomized to receive either placebo, prednisolone (10 mg twice daily), or indomethacin (50 mg twice daily). The patients treated with indomethacin tended to maintain their overall functional ability compared to those treated with placebo and to experience less pain and consume fewer analgesics. Indomethacin prolonged mean survival compared to placebo from 250 ± 28 days to 510 ± 28 days ($P < 0.05$).

The absence of a similar trend for rectal cancer could be related to the lesser power of the analyses for that tumour, resulting from the smaller numbers of subjects. In Table 2 there is a suggestion of a protective effect associated with the highest level of exposure during the period 1–6 months before diagnosis: the rate ratio was 0.80 (95% CI 0.59–1.08). Other explanations could relate to how rectal tumours manifest symptoms and are diagnosed, as well as their sensitivity to the effects of NSAIDs, which could differ from that of colonic tumours.

The dose-dependent reduction in the risk of developing colon cancer and rectal cancer associated with the dispensing of NSAIDs 11–15 years before diagnosis is unlikely to be related to chance. Selection or recall bias were very unlikely due to the study design. The effects of possible confounding by other determinants of colorectal cancer such as a high fat diet, physical inactivity, heavy beer consumption (Potter et al, 1993) and cigarette smoking (Giovannucci et al, 1994*a*, 1994*b*) remain unknown, although other studies did not detect confounding by these determinants (Rosenberg et al, 1991; Giovannucci et al, 1995). The time interval between exposure and diagnosis, the strength of the association and the consistency with the results of animal experiments (Pollard and Luckert, 1981; Birkenfeld et al, 1987) suggest to us that the protective effect is genuine.

Our results are also consistent with those from other epidemiological studies. Rosenberg et al (1991) found that exposure during the year preceding diagnosis was associated with a reduced risk of colon cancer. The duration of previous exposure did not appear to be relevant, but the effect of the timing of exposure was not evaluated. Other studies have shown a protective effect earlier in aetiologic time. Cohort studies have shown that appreciable aspirin use during the month before the inception of the cohorts was associated with a reduced rate ratio 7–10 years later (Thun et al, 1991; Gridley et al, 1993; Giovannucci et al, 1994*c*; Schreinemachers and Everson, 1994). These studies, however, measured exposure only at a single point in time: it is likely that exposure at that time was associated with exposure previous and subsequent, before the index date. The Nurses' Health Study (Giovannucci et al, 1995) measured exposure at intervals: during 1980, 1982, 1984 and 1988. Giovannucci et al (1995) found that ≥ 10 years of regular aspirin use (≥ 2 aspirin tablets per week) were required to observe a protective effect. For women exposed for ≤ 9 years the rate ratio was 0.97 (95% Cl 0.74–1.27), whereas for exposure for ≥ 10 years the rate ratio was 0.63 (95% CI 0.42–0.94).

Our results suggest that nine years of exposure to aspirin were not required but that there was a 9-year delay between exposure and the protective effect. This conclusion is supported by our finding that NSAID exposure during the periods 2–5 and 6–10 years prior to the diagnosis of either colon or rectal cancer was not associated with any significant trends towards protective effects, whereas exposure 11–15 years before was.

### CONCLUSION

We believe that the dose-dependent reduction in the rate ratios for colon cancer and rectal cancer associated with the dispensing of NSAIDs 11–15 years before the index date represents a true protective effect. The reduction in the rate ratio for colon cancer associated with NSAID exposure during the 6 months preceding the index date may represent a growth inhibiting effect of NSAIDs on already established but as yet undiagnosed cancer or a 'reverse causality' effect in which the presence of disease caused a reduction of exposure.

Our results should be considered in planning intervention trials aimed at reducing the incidence of colorectal cancer and in evaluating the results of clinical programmes recommending the use of NSAIDs to prevent colorectal cancer. Although we cannot recommend one NSAID over another, it seems evident that larger doses are likely to be more protective than smaller doses, although dose-related toxicity should also be considered. Intervention trials should be planned to last at least 10 years. Negative results from

© 1999 Cancer Research Campaign trials lasting less than 10 years (Gann et al, 1993) should not be interpreted as conclusive evidence of lack of effect – a longer duration of follow-up may be required to demonstrate a protective effect. Once the protective effect becomes apparent after a delay of about 10 years, it may persist if exposure is sustained.

### ACKNOWLEDGEMENTS

The Saskatchewan Cancer Agency and Saskatchewan Health provided the data for this study. They bear no responsibility for the analysis of the data or the interpretation of the results of this study. JP Collet is a research scholar supported by the Fonds de la Recherche en Santé du Québec. The project was funded by an operating grant from the National Health Research Development Program (NHRDP): Grant #6605-4672-503. Part of a grant for pharmacopidemiologic research from Novartis Inc. was also dedicated to the project.

### REFERENCES

Berkel HJ, Holcombe RF, Middlebrooks M and Kannan K (1996) Nonsteroidal antiinflammatory drugs and colorectal cancer. *Epidemiol Rev* **18**: 205–217

Birkenfeld S, Zaltsman YA, Krispin M, Zakut H, Zor U and Kohen F (1987) Antitumor effects of inhibitors of arachidonic acid cascade on experimentally induced intestinal tumors. *Dis Colon Rectum* **30**: 43–46

Brenner H and Gefeller O (1993) Use of the positive predictive value to correct for disease misclassification in epidemiologic studies. *Am J Epidemiol* **138**: 1007–1015

Breslow NE and Day NE (1980) *Statistical Methods in Cancer Research. Volume I – The Analysis of Case–control Studies*. International Agency for Research on Cancer: Lyon

Carson JL, Strom B, Morse ML, West SL, Soper KA, Stolley PD, et al. (1987) The relative gastrointestinal toxicity of the nonsteroidal anti-inflammatory drugs. *Arch Int Med* **147**: 1054–1059

Krogh CME (ed) (1995) *Compendium of Pharmaceuticals and Specialties*, 30th edition. Canadian Pharmaceutical Association: Ottawa

Gann PH, Manson JE, Glynn RJ, Burling JE and Hennekens CH (1993) Low-dose aspirin and incidence of colorectal tumors in a randomized clinical trial. *J Natl Cancer Inst* **85**: 1220–1224

Giovannucci E, Colditz GA, Stampfer MJ, Hunter D, Rosner BA, Willett WC et al (1994*a*) A prospective study of cigarette smoking and risk of colorectal adenoma and colorectal cancer in U.S. women. *J Natl Cancer Inst* **86**: 192–199

Giovannucci E, Rimm EB, Stampfer MJ, Colditz GA, Ascherio A, Kearney J, et al, (1994*b*) A prospective study of cigarette smoking and risk of colorectal adenoma and colorectal cancer in U.S. men. *J Natl Cancer Inst* **86**: 183–191

Giovannucci E, Rimm EB, Stampfer MJ, Colditz GA, Ascherio A and Willett WC (1994*c*) Aspirin use and the risk of colorectal cancer and adenoma in male health professionals. *Ann Intern Med* **121**: 241–246

Giovannucci E, Egan KM, Hunter DJ, Stampfer MJ, Colditz GA, Willett WC, et al (1995) Aspirin and the risk of colorectal cancer in women. *N Eng J Med* **333**: 609–614

Gridley G, McLaughlin JK, Ekbom A, Klareskog L, Adami HO, Hacker DG, et al (1993) Incidence of cancer among patients with rheumatoid arthritis. *J Natl Cancer Inst* **85**: 307–311

Lundholm K, Gelin J, Hyltander A, Lönnroth C, Sandström R and Svaninger G (1994) Anti-inflammatory treatment may prolong survival in undernourished patients with metastatic solid tumors. *Cancer Res* **54**: 5602–5606

MacMahon B and Trichopoulos D (1996) *Epidemiology. Principles and Methods*. Second Edition. Little, Brown: Boston

Marshall JR, Priore R, Graham S and Brasure J (1981) On the distortion of risk estimates in multiple exposure level case–control studies. *Am J Epidemiol* **113**: 464–473

Miettinen OS (1985) *Theoretical Epidemiology. Principles of occurrence research in medicine*. Delmar: Albany

Newcomb PA and Storer BE (1995) Postmenopausal hormone use and risk of large-bowel cancer. *J Natl Cancer Inst* **87**: 1067–1071

Paganini-Hill A, Chao A, Ross RK and Henderson BE (1989) Aspirin use and chronic diseases: a cohort study of the elderly. *Br Med J* **299**: 1247–1250

Parkin DM, Whelan SL, Ferlay J, Raymond L and Young J (eds.) (1997) *Cancer Incidence in Five Continents. Volume VII*. International Agency for Research on Cancer: Lyon

Peleg II, Lubin MF, Cotsonis GA, Clark WS and Wilcox CM (1996) Long-term use of nonsteroidal antiinflammatory drugs and other chemopreventors and risk of subsequent colorectal neoplasia. *Dig Dis Sci* **41**: 1319–1326

Pollard M and Luckert PH (1981) Effect of indomethacin on intestinal tumors induced in rats by the acetate derivate of dimethylnitrosamine. *Science* **214**: 558–559

Potter JD, Slattery ML, Bostick RM and Gapstur SM (1993) Colon cancer: a review of the epidemiology. *Epidemiol Rev* **15**: 499–545

Rawson NSB, D'Arcy C, Blackburn JL, Bowen RC, Wallace SM, Downey W, et al (1992) Epidemiologic research using linked computerized health care datafiles in Saskatchewan, Canada. Technical Report Series. In *Report #2. Psychiatric Pharmacoepidemiology Research Consortium*: Saskatoon.

Risch HA and Howe GR (1994) Menopausal hormone usage and breast cancer in Saskatchewan: a record-linkage study. *Am J Epidemiol* **139**: 670–683

Rosenberg L, Palmer JR, Zauber AG, Warshauer E, Stolley PD and Shapiro S (1991) A hypothesis: nonsteroidal anti-inflammatory drugs reduce the incidence of large-bowel cancer. *J Natl Cancer Inst* **83**: 355–358

Rothman KJ and Greenland S (1998) *Modern Epidemiology*, *2nd edition*. Lippincott–Raven: Philadelphia

Rothman KJ (1981) Induction and latent periods. *Am J Epidemiol* **114**: 253–259

SAS Institute (1992) SAS/STAT software: changes and enhancements, release 6.07. In: *SAS Technical Report P-229*. SAS Institute: Cary, NC

Schreinemachers DM and Everson RB (1994) Aspirin use and lung, colon and breast cancer incidence in a prospective study. *Epidemiology* **5**: 138–146

Thun MJ, Namboodiri MM and Health CW (1991) Aspirin use and reduced risk of fatal colon cancer. *N Eng J Med* **325**: 1593–1596

*© 1999 Cancer Research Campaign*
